# Understanding the Role of Reading and Oral Language Skills Growth in Overcoming Reading Comprehension Difficulties

**DOI:** 10.3390/bs16010090

**Published:** 2026-01-08

**Authors:** Apostolos Kargiotidis, George Manolitsis

**Affiliations:** Department of Preschool Education, University of Crete, 74150 Rethymno, Greece; a.kargiotidis@edc.uoc.gr

**Keywords:** oral language, reading comprehension difficulties, morphological awareness, phonological awareness, vocabulary, longitudinal study

## Abstract

The present longitudinal retrospective study examined in a sample of 123 Greek-speaking children whether the raw score growth in a broad range of oral language and reading skills from Grade 1 to Grade 3 differs among children with persistent reading comprehension difficulties (pRCD; *N* = 49) identified in Grade 3, those exhibiting a resolving tendency of RCD (rRCD; *N* = 16), and typically developing (TD; *N* = 58) children. Children were classified into the respective groups, based on their performance on standardized reading comprehension measures in Grades 1, 2, and 3. They were, also, assessed on phonological awareness, rapid automatized naming (RAN), morphological awareness, vocabulary, word reading accuracy, word reading fluency, and text-reading fluency across the three Grades. Mixed ANOVAs showed that children with pRCD displayed slower growth in morphological awareness, word reading fluency, and text-reading fluency than the other two groups. Children with rRCD did not differ from TD children on these measures, but they exhibited a higher growth on RAN. Both groups of children with RCD outperformed TD children on the growth of phonological awareness and word reading accuracy, whereas no group differences revealed in vocabulary. Our results suggest that more rapid gains in morphological awareness, RAN, word reading fluency, and text-reading fluency over time might be associated with a resolving tendency of reading comprehension difficulties, providing valuable insights for intervention policy.

## 1. Introduction

Reading comprehension—the ability to extract meaning from written text—is the ultimate goal of learning to read (Nation, 2019). It is considered as a complex process that requires the integration of well-developed linguistic and decoding skills (Gough & Tunmer, 1986; Hoover & Gough, 1990). According to the Lexical Quality Hypothesis, the oral language domains of phonology, semantics, and morphosyntax, together with orthographic knowledge, support the formation of high-quality lexical representations of words, which, in turn, facilitate reading comprehension (C. Perfetti, 2007). In line with that, empirical evidence has indicated that oral language skills account for a substantial proportion of the variance in reading comprehension (Hjetland et al., 2019). They contribute to reading comprehension either directly (e.g., morphological awareness and vocabulary) (Fernandes et al., 2017; Manolitsis et al., 2019), or indirectly (e.g., phonological processing skills), by underpinning the development of decoding skills (Hjetland et al., 2020; Hulme et al., 2015). On the other hand, according to the Verbal Efficiency Theory, well-developed decoding skills can release valuable cognitive resources, such as working memory, that can be allocated to the comprehension of written text (C. A. Perfetti, 1985).

However, although reading comprehension might develop relatively effortlessly for some children, a considerable proportion of them (around one in every three fourth-grade students in the U.S.) faces reading comprehension difficulties (RCD) (Spencer et al., 2014). Findings from the recent Program for International Student Assessment (PISA; OECD, 2023) revealed a notable decline in the reading comprehension performance of 15-year-old students in most of the 81 participating countries, compared to the 2018 results. Of particular concern, was the performance of Greek students which was significantly below the OECD average (i.e., OECD average score = 476 and Greece average score = 438). To achieve academic success and become competent members of society, children should be able to read and understand a variety of texts. Consequently, the need for evidence-based interventions to support struggling readers is of vital importance. Empirical research has systematically shown that deficits in core oral language skills and/or inefficient reading skills significantly increase the risk of RCD (Cain, 2016; Capin et al., 2022; Grigorakis et al., 2022).

However, there is a paucity of longitudinal research examining whether changes in oral language and reading skills over time are associated with concurrent gains in reading comprehension of children with RCD. Understanding these patterns could be rather beneficial for developing targeted and developmentally appropriate interventions to prevent the emergence of RCD early (Hjetland et al., 2017). Thus, the aim of the present study was to examine whether the raw score growth in a broad range of oral language and reading skills differs between children with persistent RCD, children with a resolving tendency of RCD, and typically developing (TD) children from Grade 1 to Grade 3.

### 1.1. Oral Language Skills and Reading Comprehension Development

A considerable amount of research has systematically shown that oral language skills are an important predictor of reading comprehension already from the first elementary grades (Foorman et al., 2015; Kendeou et al., 2009; Manolitsis et al., 2019; Protopapas et al., 2013). Moreover, Hjetland et al. (2019) found that children’s oral language skills at the age of seven do not only concurrently predict their reading comprehension levels, but also the growth rate of reading comprehension until the age of nine.

In more detail, studies in different orthographies increasingly emphasize the important role of morphological awareness on reading comprehension development, already from the first stages of learning to read (Desrochers et al., 2018; Y. G. Kim et al., 2019; Manolitsis et al., 2017; Müller & Brady, 2001; Sparks & Metsala, 2023). Morphemes are the smallest meaningful units of language that carry phonological, semantic, and syntactic information (Mahony et al., 2000) and they are largely involved in the word formation process, the syntactic connection of words within a sentence, and word recognition. Morphological awareness enables children to segment morphologically complex words into their constituent morphemes, thereby enhancing their ability to decode them, extract meaning, and perceive the syntactic structure of sentences, all of which facilitate reading comprehension (Levesque et al., 2017). Consequently, studies have indicated that children with poor morphological awareness are at greater risk of presenting with RCD (Adlof & Catts, 2015; Tong et al., 2013). In line with that, Grigorakis et al. (2022) found that Greek-speaking children with morphological awareness deficits in Grade 1 are more likely to present with RCD in Grade 2, despite adequate reading fluency. The association between morphological deficits and RCD could be described as a vicious cycle. In particular, children with RCD are expected to devote less effort and time on reading material, which results in fewer opportunities to read and learn morphologically complex words (MacKay et al., 2017; Tong et al., 2013). On the other hand, children with poor morphological awareness might not be able to exploit all the semantic cues encompassed in morphemes to fully comprehend a text because they might struggle to understand how the variation in the lexical form of words affects their meaning (Kieffer, 2014).

Vocabulary is another semantically oriented oral language skill that is actively involved in reading comprehension development (Kargiotidis et al., 2022; Muter et al., 2004; Verhoeven & van Leeuwe, 2008). Empirical research has indicated that limited vocabulary knowledge is also a risk factor of RCD (Catts et al., 2006). For example, in the retrospective study of Nation et al. (2010) results showed that eight-year-old children with RCD significantly lagged behind in vocabulary knowledge compared to their TD peers two years earlier. Similarly, Catts et al. (2016) found that receptive vocabulary in kindergarten significantly predicts RCD at the end of Grade 3. Moreover, in the longitudinal study of Cain and Oakhill (2011) results showed that the growth rate of vocabulary is significantly lower for children with specific RCD than for children with good reading comprehension. In the Greek orthography, Rothou (2021) found that third-grade children with vocabulary deficits are more likely to struggle to comprehend written text, despite adequate decoding skills. In line with that, the longitudinal study of Grigorakis et al. (2022) showed that first-grade children with poor vocabulary knowledge are at greater risk to experience specific RCD in second grade.

According to Hjetland et al. (2019, 2020), oral language skills may also indirectly affect reading comprehension. Specifically, their results showed that early phonological processing skills, such as phonological awareness and rapid automatized naming (RAN), can provide the foundations for the development of decoding skills, which, in turn, contribute to reading comprehension. Several studies examining the role of phonological awareness in reading comprehension have also corroborated that its effects are fully mediated by word decoding skills (Georgiou et al., 2009; Høien-Tengesdal, 2010; Kargiotidis et al., 2022). Furthermore, Hulme et al. (2015) found that deficits in the phonological processing skills of children at familial risk of dyslexia compromise the development of their decoding skills and henceforth reading comprehension. However, research findings in highly transparent orthographies, like Greek, have indicated that children with reading difficulties manage to adequately develop their phonological processing skills and approach typical levels (Kargiotidis et al., 2021; Schmidt et al., 2021). Thus, it could be expected that the initial phonological deficits of children with RCD will tend to diminish over time.

The findings of Hjetland et al. (2019), indicating that early oral language skills can predict later growth rate of reading comprehension, align with the results of intervention studies demonstrating that gains in oral language skills might foster reading comprehension of children with RCD. In particular, in a randomized controlled trial, Clarke et al. (2010) found that an oral language intervention might result in significant improvements in reading comprehension of children with RCD which are fully or partially mediated by gains in vocabulary knowledge. Interestingly, children in the oral language training group show greater gains in reading comprehension than children in a text-comprehension training group or in a combined training group. Also, Goodwin and Ahn (2010) in their meta-analysis of the results from 17 intervention studies found that morphological instruction may result in significant improvements in both morphological awareness and reading comprehension of children with literacy difficulties. Another meta-analysis conducted by McArthur et al. (2018) revealed that phonological awareness and phonics instruction may slightly improve reading comprehension of children with word reading difficulties. Moreover, Wolfsperger and Mayer (2024) found that children who participated in a RAN training demonstrated significant gains in their RAN performance, as well as on reading comprehension at both word and sentence level. Improvements in RAN performance through targeted RAN interventions have also been associated with corresponding gains in reading fluency and accuracy (Margheri et al., 2025; Pecini et al., 2019), which serve as foundational skills for reading comprehension development. Thus, the existing research evidence from intervention studies provides a preliminary basis for the hypothesis that oral language growth might be associated with gains in reading comprehension for children with RCD.

To sum up, a considerable body of research underlines the essential role of oral language skills in the development of reading comprehension from the earliest stages of formal reading instruction. Cross-sectional (e.g., Tong et al., 2013) and longitudinal (e.g., Grigorakis et al., 2022) studies demonstrate that deficits in these domains increase the risk of RCD. Additionally, research findings from intervention studies (e.g., Clarke et al., 2010; Goodwin & Ahn, 2010) demonstrate that oral language gains, particularly through morphological and vocabulary instruction, can foster reading comprehension development. Collectively, this evidence provides an empirical foundation for the hypothesis that promoting oral language development may serve as an effective pathway to prevent or mitigate RCD in children.

### 1.2. The Role of Word-Level and Text-Level Reading Skills on Reading Comprehension Development

According to the Simple View of Reading, oral language comprehension alone is not sufficient for reading comprehension unless it is accompanied by well-developed word reading skills (Hoover & Gough, 1990). Research findings have indicated that word reading skills can predict reading comprehension both concurrently and over time with their influence being stronger than that of oral language skills during the early grades (Catts et al., 2015; Muter et al., 2004). In a longitudinal study of children at familial risk of dyslexia, Hulme et al. (2015) found that early word reading accuracy provides the foundation for the development of reading comprehension three years later. However, based on the Verbal Efficiency Theory, children must be able to read words both accurately and fluently to comprehend written text because slow and effortful word reading ties up valuable cognitive resources, such as working memory, that are necessary for reading comprehension (C. A. Perfetti et al., 1996). Indeed, Capin et al. (2022) found that in a sample of children in Grades 1–4 with language and reading comprehension difficulties the majority of them exhibited significant difficulties in word reading fluency. Moreover, Kang and Shin (2019) indicated that word reading fluency is a more consistent predictor of reading comprehension of fourth-grade children with RCD than word reading accuracy. Similarly, the longitudinal study of Cain and Oakhill (2011) revealed that children with specific RCD and children with good reading comprehension do not differ in the growth rate of word reading accuracy between the ages of 8 and 16. Consequently, word reading fluency is regarded as a key determinant of reading comprehension in upper elementary grades.

Moreover, the meta-analysis of Florit and Cain (2011) revealed that for novice readers in transparent orthographies, like Greek, reading fluency tends to be more closely associated with reading comprehension than reading accuracy. In line with that, studies in transparent orthographies have shown that even children with reading difficulties manage to adequately develop accurate word reading (Aro & Wimmer, 2003; Landerl & Wimmer, 2008) rendering word reading fluency a more trustworthy predictor of RCD than word reading accuracy. Furthermore, the results of a recent longitudinal study (Kargiotidis et al., 2025), that examined an extended version of the SVR model for the prediction of RCD, showed that children’s RCD in Grade 3 are significantly predicted by word reading fluency in Grade 2 but not word reading accuracy.

While word reading fluency remains a foundational component of reading comprehension, recent empirical research has increasingly emphasized the significance of text-reading fluency in shaping reading comprehension outcomes (Y. G. Kim, 2015). Although it is closely associated with word reading fluency, text-reading fluency is considered as a separate construct that captures children’s ability to read fast and accurately a connected text (Y. G. Kim & Wagner, 2015). Research findings have indicated that text-reading fluency accounts for additional variance in reading comprehension beyond the effects of word reading and oral language skills (Jenkins et al., 2003; Y.-S. Kim et al., 2012). In particular, in a longitudinal study from Grades 1 to 4, Y. G. Kim and Wagner (2015) found that after Grade 1 text-reading fluency completely mediates the association of both word reading fluency and listening comprehension with reading comprehension. The more decisive role of text-reading fluency in the development of reading comprehension at higher grade levels is more evident in studies involving children with RCD. Specifically, Clemens et al. (2017) found that the majority of students with RCD from sixth through eighth grade shows deficits in text-reading fluency and/or vocabulary. Also, according to Eason et al. (2013), poor comprehenders between the ages 10 and 14 show similar word reading fluency levels with average readers but lag behind them on text-reading fluency. Finally, the findings of Cutting et al. (2009) confirmed the distinction between word-reading and text-reading fluency by indicating that children with specific RCD perform similarly on word reading fluency to TD children but are outperformed on text-reading fluency.

However, although empirical research has highlighted the critical role of word reading skills and text-reading fluency on reading comprehension development, there is a paucity of longitudinal studies investigating whether improvements in reading comprehension of children with RCD are associated with corresponding changes in their reading skills. Moreover, research findings from reading intervention studies are quite inconclusive. For example, in a synthesis of fluency intervention research from 2001 to 2014, Stevens et al. (2016) found that this kind of reading intervention—and especially repeated reading—can significantly contribute to the development of word reading skills and reading comprehension of children with learning disabilities from kindergarten to the fifth grade. In essence, their results demonstrate that enhancing automaticity in text processing can facilitate the development of reading comprehension of children with learning disabilities. Similarly, Wolff (2011) indicated that an intensive multicomponent intervention that targets phoneme awareness/decoding, reading comprehension strategies, and fluency training in nine-year old children with reading difficulties might result in significant gains in reading fluency and reading comprehension. Moreover, the meta-analysis of Zimmermann et al. (2019) demonstrated that non-repetitive reading fluency interventions has significant effects on reading comprehension of K-12 students with reading difficulties. In contrast, Wanzek et al. (2020) found that although fourth-grade children with RCD who receive an intensive multicomponent reading intervention outperformed the control group on word reading skills, they do not differ in the growth rate of reading comprehension. Also, in the meta-analysis of Suggate (2014) results showed that interventions that target reading fluency might be less effective for reading comprehension development than mixed interventions that target comprehension and a phonics or phonemic awareness component.

The accumulated research evidence demonstrates that while word reading accuracy provides an initial foundation for reading comprehension, it is the development of word reading fluency and, increasingly, text-reading fluency that appears to determine how effectively children can allocate cognitive resources to comprehend connected text. Moreover, intervention studies (e.g., Zimmermann et al., 2019) indicate that improvements in reading fluency can trigger reading comprehension development, though the evidence is mixed, and fluency interventions alone may not guarantee growth in comprehension.

Overall, a substantial body of research (e.g., Catts et al., 2015; Hjetland et al., 2019) has already documented the associations of oral language, word-level and text-level reading skills with reading comprehension, as well as their predictive relations. However, it remains less clear how changes in oral language and reading skills over time are linked to within-child changes in reading comprehension. Moreover, despite a considerable number of intervention studies (e.g., Clarke et al., 2010; Zimmermann et al., 2019), there is a dearth of longitudinal evidence examining whether children who show a resolving tendency of RCD differ from those with persistent RCD in the rate of change across several oral language and reading domains.

### 1.3. The Present Study

The purpose of the present longitudinal retrospective study was to examine whether raw score growth in a broad range of oral language and reading skills from Grade 1 to Grade 3 differs among children with a profile of persistent reading comprehension difficulties (pRCD) in Grade 3, those exhibiting a resolving tendency of RCD (rRCD), and typically developing (TD) children. Our results aim to clarify whether a more increased growth on specific oral language and reading skills is associated with a more rapid development of reading comprehension for children with RCD. The following research questions and respective hypotheses were addressed by the present study:

(1) Does the raw score growth of oral language skills from Grade 1 to Grade 3 differ among children with a profile of pRCD, those with rRCD, and TD children in Grade 3?

Several studies have highlighted the crucial role of vocabulary and morphological awareness on the development of reading comprehension (Fernandes et al., 2017; Manolitsis et al., 2019; Muter et al., 2004; Verhoeven & van Leeuwe, 2008). Furthermore, previous research findings have demonstrated that children with deficits in these two semantically oriented oral language skills are more likely to present with RCD (Adlof & Catts, 2015; Catts et al., 2006; Grigorakis et al., 2022; Tong et al., 2013). Finally, intervention studies have revealed that fostering children’s morphological awareness and vocabulary knowledge may result in significant advances on reading comprehension (Clarke et al., 2010; Goodwin & Ahn, 2010). Therefore, we hypothesized that children with rRCD will show a more increased raw score growth on morphological awareness and vocabulary from Grade 1 to Grade 3 than children with pRCD (H1).

Furthermore, the influence of phonological processing skills on reading comprehension development is considered as more indirect than that of morphological awareness and vocabulary, whose effects are fully mediated by word reading skills (Hulme et al., 2015; Kargiotidis et al., 2022). Also, research findings in highly transparent orthographies, like Greek, have shown that even children with reading difficulties manage to adequately develop their phonological processing skills and approach typical levels (Kargiotidis et al., 2021; Schmidt et al., 2021). Finally, research evidence shows that phonological awareness and phonics instruction (McArthur et al., 2018) and RAN training (Wolfsperger & Mayer, 2024) can foster reading comprehension development. Thus, we hypothesized that both children with pRCD and children with rRCD will show a faster raw score growth on phonological processing skills from Grade 1 to Grade 3 than TD children (H2).

(2) Does the raw score growth of word reading skills and text-reading fluency from Grade 1 to Grade 3 differ among children with a profile of pRCD, those with rRCD, and TD children in Grade 3?

Previous research findings in transparent orthographies have underlined the prominent role of word reading fluency in the development of reading comprehension (Florit & Cain, 2011). In contrast, the role of word reading accuracy is considered to be less determinative for the emergence of RCD than that of word-reading fluency (Kargiotidis et al., 2025) with children with reading difficulties showing adequate word reading accuracy already from the first elementary grades (Aro & Wimmer, 2003; Landerl & Wimmer, 2008). Therefore, we hypothesized that both children with pRCD and those with rRCD in Grade 3 will show a faster raw score growth on word reading accuracy form Grade 1 to Grade 3 than TD children (H3). Moreover, we assumed that children with pRCD in Grade 3 will show a slower raw score growth on word-reading fluency from Grade 1 to Grade 3 than the other two groups of children (H4).

Finally, research findings have indicated that after the first grade (Y. G. Kim & Wagner, 2015) and as children are getting older (Clemens et al., 2017; Eason et al., 2013) text-reading fluency plays a more decisive role in the development of reading comprehension, with children who have deficits in text-reading fluency being at greater risk of RCD. Therefore, we hypothesized that children with pRCD will show a slower raw score growth on text-reading fluency than children with rRCD and TD children (H5).

## 2. Materials and Methods

### 2.1. Participants

The participants of the present longitudinal retrospective study were 123 Greek-speaking children (62 girls; mean age = 79.15 months; SD = 3.65, at the first time of measurement in grade 1) from public mainstream elementary schools in Heraklion, Greece. Participants were initially selected from a sample of 240 first-grade children (see (Kargiotidis et al., 2021) for more details about participant recruitment procedure), based on whether they met the criteria to be classified in one of the three literacy groups. Then, they were followed up until the end of Grade 3. All children had written parental consent, typical non-verbal ability (i.e., Raven’s score above 70) and no formal diagnosis of any intellectual, neurodevelopmental, or sensory disorder.

Regarding language and reading instruction in the Greek educational system, instructional emphasis varies across grades. In Grade 1, there is a strong emphasis on phonics through an analytic-synthetic approach, focusing on the acquisition of letter-sound correspondences and decoding skills. From Grade 2 onward, instruction increasingly incorporates the explicit teaching of morphological rules, along with the development of reading fluency, spelling accuracy, and writing skills. By Grade 3, instruction continues to build on these foundations, with greater emphasis on morphological awareness and reading comprehension.

### 2.2. Measures

#### 2.2.1. Non-Verbal Intelligence

Non-verbal intelligence was assessed with the Greek standardization of the Raven’s Colored Progressive Matrices (Raven, 1956; Sideridis et al., 2015). Children were instructed to choose among six alternatives the one that best matched to a presented matrix with a missing part. Cronbach’s alpha in the standardization sample was 0.90 (Sideridis et al., 2015).

#### 2.2.2. Morphological Awareness

A *Word Analogy* and a *Compound Word Production* task (see Manolitsis et al., 2017) was used to assess morphological awareness. The Word Analogy task comprises 26 items examining inflectional and derivational morphological awareness (i.e., twelve items for inflectional morphology and fourteen items for derivational morphology). Initially, children were orally presented with a pair of words in which they had to recognize the morphological association. Next, they were instructed to apply the same morphological association to orally complete a second pair of words (e.g., /ʝa’tri/: /ʝa’tros/:: /ae’ti/: (/ae’tos/)— “doctors”: “doctor”:: “eagles”: (“eagle”)) and /ku’no/: /’kunima/:: /xti’po/: (/’xtipima/)—“I shake”: (the) “shaking”:: “I hit”: ((the) “hitting”)). Four practice items preceded formal testing, two for each morphological condition. It should be mentioned that in Grades 1 and 2 only the first 20 items were administered to children. Therefore, the maximum score a child could achieve was 20. The last 6 items were administered in Grade 3 to avoid ceiling effects for TD children (i.e., the maximum score in Grade 3 was 26). Testing was terminated after six consecutive errors. Cronbach’s alpha in the current sample was 0.93 for Grade 1, 0.92 for Grade 2, and 0.86 for Grade 3.

The Compound Word Production task consisted of 21 items measuring lexical compounding awareness. Children were orally presented a pair of words and they had to transform them into stems to orally produce a compound word (e.g., “How could we call?” /tis ‘γatas to ‘ðromo/ “the cat’s road” > (/γa’toðromo/ “catroad”). Similarly to the Word analogy task, we administered only the first 15 items in Grades 1 and 2. The last 6 items were administered in Grade 3 to avoid ceiling effects. Therefore, the maximum score a child could achieve in Grades 1 and 2 was 15, and in Grade 3 it was 21. Testing was terminated after four consecutive errors. Cronbach’s alpha in the current sample was 0.90 for Grade 1, 0.90 for Grade 2, and 0.91 for Grade 3.

In the subsequent statistical analyses, we calculated the composite score for MA in each one of the three Grades by adding the raw scores of the respective component tasks. Cronbach’s alpha for the composite variable was 0.94 for Grade 1 and Grade 2, and 0.93 for Grade 3.

#### 2.2.3. Vocabulary

The “*Vocabulary*” subscale of the Greek standardization of the Wechsler Intelligence Scale—Fifth Edition (WISC-V^GR^; Stogiannidou, 2017) was used to assess vocabulary. Initially, children were asked to name what was depicted in four pictorial items. Each correct answer was scored with 1 point. In the next 25 items, they were instructed to verbally define an orally presented word. Each answer was scored with 0, 1 or 2 points based on the quality of the definition. Testing was terminated after three consecutive answers scored with 0. In the standardization sample the average split-half reliability coefficient (odd versus even items) across all age groups was 0.83 (Stogiannidou, 2017).

#### 2.2.4. Phonological Awareness

Three tasks were used to assess phonological awareness: *Elision with real words, Elision with pseudowords*, and *Blending*. Each of the Elision tasks (see Manolitsis et al., 2019) included four practice items and thirty-six experimental items distributed in six blocks of six items in ascending order of difficulty. Initially, each item was orally presented to children and they were asked to repeat it. Then, they were instructed to remove a specific onset, rime, syllable, or phoneme from the presented item and say what remained. It should be noted that in Grades 1 and 2 only the items from the first 4 blocks were administered to children (i.e., 24 items in total). Therefore, the maximum score a child could achieve was 24. The items of the fifth and the sixth block were administered in Grade 3 to prevent ceiling effects (see (Kieffer & Lesaux, 2012) for a similar practice), especially for TD children. In all Grades, testing was discontinued after four errors in a particular block. Cronbach’s alpha reliability coefficient in our sample was for the *Elision with real words* task 0.94 for Grade 1, 0.91 for Grade 2, and 0.92 for Grade 3 and for the *Elision with pseudowords* task 0.95 for Grade 1, 0.90 for Grade 2, and 0.93 for Grade 3.

The Blending task included four practice items and thirty-six test items in increasing order of difficulty (see Manolitsis & Georgiou, 2015). Children were instructed to listen to a sequence of individual sounds and then to combine them to create a whole word. In the first three items they had to join together two syllables, in the next five an onset and a rime, and in the remaining items, they were asked to merge separate phonemes, ranging from two to thirteen. Similarly to the Elision tasks, we administered only the first 28 items in Grades 1 and 2. The remaining 8 items were administered in Grade 3 to avoid ceiling effects. Therefore, the maximum score a child could achieve in Grades 1 and 2 was 28, and in Grade 3 it was 36. Cronbach’s alpha reliability coefficient in our sample was 0.90 for Grade 1, 0.88 for Grade 2, and 0.90 for Grade 3. In all grades, testing was terminated after four consecutive errors.

In the subsequent statistical analyses, we calculated the composite score for PA in each one of the three Grades by adding the raw scores of the respective component tasks. Cronbach’s alpha for the composite variable was 0.97 for Grade 1, 0.95 for Grade 2, and 0.96 for Grade 3.

#### 2.2.5. Rapid Automatized Naming

To assess RAN, a *Color naming* task and a *Digit naming* task (Landerl et al., 2019) were used in all Grades. In the Color naming task, children were asked to name from left to right as quickly and precisely as possible the names of four recurring colors (yellow, green, blue, and red). Colors were visually presented and semi-randomly arranged in four rows of six in two separate cards. Initially, children were tested in a practice trial to ensure that they were aware of the four colors’ names. The corresponding names of the four colors in Greek are /*kitrino*/ for yellow, /*prasino*/ for green, /*ble*/ for blue, and /*kokino*/ for red. A participant’s score was the average time in milliseconds to name both cards.

In the Digit naming task, children had to name from left to right as quickly and accurately as possible the names of four recurring digits (5, 4, 7, and 2). Digits were visually presented and semi-randomly arranged in four rows of six in two separate cards. Initially, children were tested in a practice trial to ensure that they were aware of the four digits’ names. The corresponding names of the four digits in Greek are /*pende*/ for five, /*tesera*/ for four, /*efta*/ for seven, and /*ðio*/ for two. A participant’s score was the average time in milliseconds to name both cards.

#### 2.2.6. Word Reading Accuracy

Word reading accuracy was assessed with the “*Word Decoding*” subscale of a Greek standardized reading measure (Padeliadu et al., 2019). Children were asked to read a list of 57 words, arranged in ascending order of difficulty, with no time constraint. Testing was discontinued after five consecutive errors. Cronbach’s alpha in the current sample was 0.96 for Grade 1, 0.94 for Grade 2, and 0.92 for Grade 3.

#### 2.2.7. Word Reading Fluency

The *Word Reading Efficiency* (WRE) scale adapted in Greek by Georgiou et al. (2008) following the Test of Word Reading Efficiency (TOWRE; Torgesen et al., 1999) was used to assess word reading fluency. Children were instructed to read as quickly and accurately as possible a list of 104 words, equally distributed in four columns in ascending order of difficulty, in 45 s. The total score for each child was the number of correctly read words within the time limit. Test–retest reliability coefficient has been estimated at 0.94. In addition, in the current sample the inter-correlation coefficients among the three Grades ranged from 0.82 to 0.87.

#### 2.2.8. Text Reading Fluency

Text-reading fluency was assessed using the “*Text-Reading Fluency*” subscale from a Greek standardized reading measure (Padeliadu et al., 2019). Children were instructed to read a 247-word passage about an ancient Greek myth as quickly and accurately as possible within 1 min. The total score for each child was the number of the correctly read words within the time limit. The test–retest reliability reported in the standardization study was 0.98 (Padeliadu et al., 2019). Also, in our sample the inter-correlation coefficients among the three Grades ranged from 0.77 to 0.86.

#### 2.2.9. Reading Comprehension

In Grade 1, reading comprehension was assessed with the Greek standardized “*Reading and Sentence Completion Test*” (Porpodas, 2008), which is a sentence-completion test that includes sentences of increasing difficulty, in terms of word number and semantic information. It consists of 16 sentences, each of which is missing a word, and children were instructed to choose among three options the word that best completed each sentence without any time constraint. Cronbach’s alpha in the current sample was 0.94. In Grades 2 and 3, reading comprehension was assessed with the Greek standardized “*Screening Test of Reading Ability*” (Tafa, 1995), which is also a sentence-completion test that consists of 42 sentences with one missing word each. Children were instructed to choose among four alternative words the one that correctly completed each sentence within 40 min. Cronbach’s alpha in the current sample was 0.89 for Grade 2 and 0.92 for Grade 3.

### 2.3. Procedure

Trained research-assistants (postgraduate students of psychology or education) assessed children in all three Grades. Specifically, non-verbal intelligence and oral language skills were measured in two 20 min individual sessions in the middle of Grade 1. At the end of Grade 1, word reading accuracy, word reading fluency, and text reading fluency were assessed in a 10 min individual session whereas reading comprehension was measured in a group-session of 10 children in each group. At the beginning of Grade 2, word reading accuracy, word reading fluency, and text reading fluency were assessed again in a 10 min individual session while reading comprehension was measured in a 40 min group session of 10 children in each group. In the middle of Grade 2, children were assessed on oral language skills in two 20 min individual sessions. Finally, at the middle of Grade 3 oral language skills were assessed again in two 20 min individual sessions whereas reading skills were measured at the end of Grade 3 in a 10 min individual session for word reading accuracy, word reading fluency, and text reading fluency and a 40 min group session for reading comprehension. The present study was approved by the Greek Ministry of Education and the Research Ethics Committee of the Department of Preschool Education of the University of Crete.

### 2.4. Statistical Analysis

Prior to statistical analyses, children were classified into three literacy groups based on their performance on the standardized reading comprehension measures in the first three elementary grades. The selected classification criteria are consistent with those from previous research examining reading comprehension difficulties (e.g., Bailey et al., 2016; Kelso et al., 2020). In particular, those children who performed equally or below the 25th percentile in all three grades were classified in the group with persistent RCD (*N* = 49; 26 girls). On the other hand, those children who performed equally or below the 25th percentile in Grades 1 and 2 but equally or above the 37th percentile in Grade 3 were classified into the group with a resolving tendency of RCD (*N* = 16; 9 girls). The TD group (*N* = 58; 27 girls) consisted of those children who performed equally or above the 37th percentile in all three grades. Moreover, the composite scores for PA and MA in each one of the three Grades were calculated by adding the raw scores of the respective component tasks whereas the composite score for RAN was the average score of children’s mean performance on the RAN Digits and RAN Colors tasks. Finally, no missing values or extreme outliers were detected.

Regarding the statistical analyses, a series of one-way analyses of variance (ANOVAs) was conducted to examine the between-group differences on non-verbal intelligence in Grade 1 and oral language and reading skills across the three Grades. Next, a series of mixed ANOVAs was performed to assess whether the three groups differed from each other on the raw score growth of oral language and reading skills during the first three elementary grades. ANOVAs included one within-subjects factor of Grade (Grade 1 vs. Grade 2 vs. Grade 3) and one between-subjects factor of literacy group (pRCD vs. rRCD vs. TD). Finally, when a significant interaction of grade X literacy group was detected, it was further investigated through a series of one-way ANOVAs with post hoc Bonferroni comparisons to determine between which groups and for which time intervals (i.e., Grade 1 to Grade 2, Grade 2 to Grade 3, or Grade 1 to Grade 3) there was a significant difference in the raw score growth of oral language and reading skills.

## 3. Results

### 3.1. Preliminary Analyses

Descriptive statistics for each literacy group for non-verbal intelligence in Grade 1 and oral language and reading skills across the three Grades are presented in Table 1 and Table 2. Regarding reading skills, results from one-way ANOVAs revealed statistically significant differences among the three groups of children across the three Grades. In particular, post hoc Bonferroni comparisons revealed that children with persistent RCD and those with a resolving tendency of RCD performed significantly lower than TD children on all reading measures across Grades 1 to 3 (Table 1). However, children with persistent RCD lagged behind children with a resolving tendency of RCD in word reading accuracy in Grades 2 and 3 and on word reading fluency, text-reading fluency, and reading comprehension in Grade 3.

Similarly, results from one-way ANOVAs showed statistically significant differences in all oral language skills among the three groups of children across the three Grades. Specifically, post hoc Bonferroni comparisons revealed that children with pRCD and children with rRCD lagged behind TD children on all oral language skills in all Grades (Table 2). Also, children with pRCD performed significantly lower on PA in Grades 2 and 3 and MA and vocabulary in Grade 3 than children with rRCD. Interestingly, results showed an inverse pattern regarding the differences in the magnitude of the effect sizes of phonologically and semantically oriented skills from Grade 1 to Grade 3 between the three groups. In particular, the effect sizes of PA presented a gradual decline, whereas those of MA and vocabulary exhibited incremental increase, potentially indicating a developmental shift in their respective roles.

Finally, although our results showed that children with pRCD and those with rRCD scored significantly lower than TD children on non-verbal intelligence in Grade 1, no significant differences were found on non-verbal intelligence levels between the two groups with reading comprehension difficulties. Therefore, the observed differences in reading comprehension growth between children with pRCD and those with rRCD could not be attributed to variations in non-verbal intelligence. On the other hand, the finding that poor comprehenders exhibited a lower mean score in non-verbal intelligence compared to TD children is not surprising (Nation et al., 2002). This difference, however, does not imply that their intelligence falls below normal range.

### 3.2. Differences in the Raw Score Growth of Oral Language and Reading Skills

**Figure 1:**
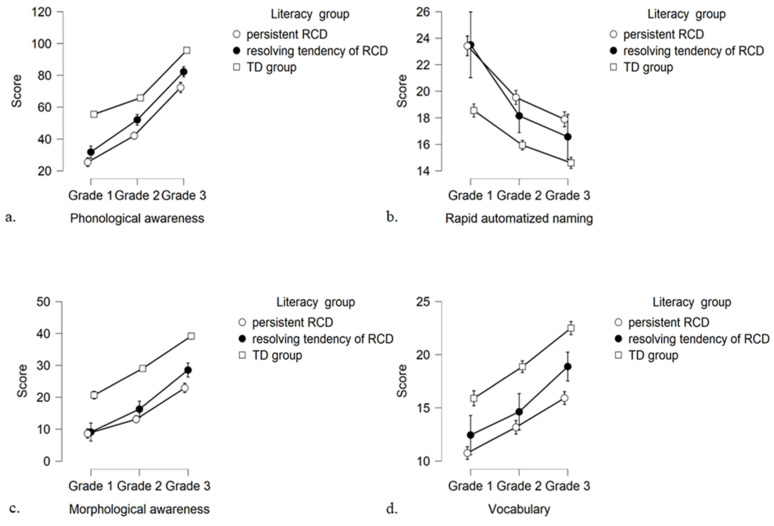
Means and confidence intervals (95%) of scores on Phonological awareness (**a**), Rapid automatized naming (**b**), Morphological awareness (**c**), and Vocabulary (**d**) in the first three grades between the three literacy groups.

Results from mixed ANOVAs revealed significant main effects of grade (η^2^_ρ_ = 0.55–0.88) and literacy group (η^2^_ρ_ = 0.28–0.68) for all oral language skills. Furthermore, results revealed a significant interaction effect of grade X literacy group for phonological awareness, *F*(3.66, 219.65) = 5.45, *p* < 0.01, η^2^_ρ_ = 0.08, morphological awareness, *F*(3.65, 218.82) = 4.97, *p* < 0.01, η^2^_ρ_ = 0.08, and RAN, *F*(3.20, 191.93) = 4.43, *p* < 0.01, η^2^_ρ_ = 0.07^1^, but not for vocabulary, *F*(4, 240) = 1.54, *p* > 0.05, η^2^_ρ_ = 0.03; A closer examination of Figure 1 shows that the significant interactions might be due to the different raw score growth of MA, PA, and RAN among the three groups of children from Grade 1 to Grade 3. Follow-up one-way ANOVAs with post hoc Bonferroni comparisons were conducted to examine further these interactions by computing the mean difference scores for MA, PA, and RAN across the three groups of children over the three time intervals (i.e., Grade 1 to Grade 2, Grade 2 to Grade 3, and Grade 1 to Grade 3) (see Table 3). Results revealed that children with pRCD and children with rRCD showed significantly higher mean difference scores on PA from Grade 1 to Grade 2 and from Grade 1 to Grade 3 than TD children. Also, children with pRCD showed significantly lower mean difference scores on MA from Grade 1 to Grade 2 than TD children and from Grade 1 to Grade 3 than children with rRCD and TD children. Finally, children with rRCD showed significantly higher mean difference scores on RAN than TD children from Grade 1 to Grade 2 and from Grade 1 to Grade 3.

**Figure 2:**
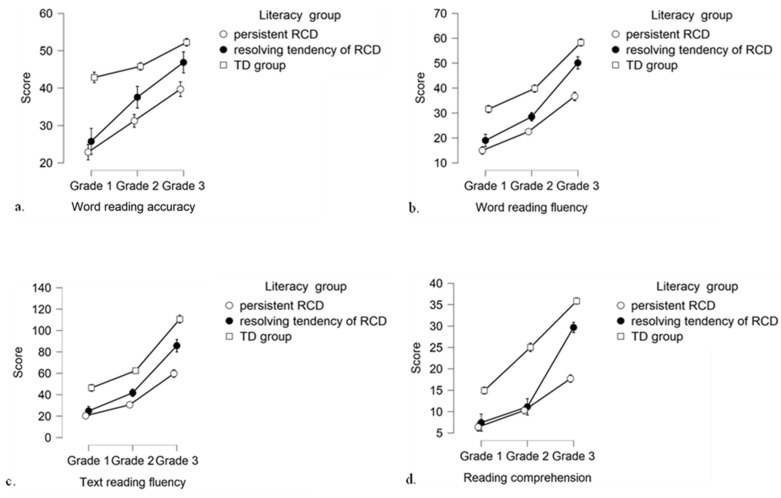
Means and confidence intervals (95%) of scores on Word reading accuracy (**a**), Word reading fluency (**b**), Text reading fluency (**c**) and Reading comprehension (**d**) in the first three grades between the three literacy groups.

Another series of mixed ANOVAs was conducted to examine the differences in the raw score growth of reading skills among the three groups of children. Results revealed a significant main effect of grade (η^2^_ρ_ = 0.60–0.87) and literacy group (η^2^_ρ_ = 0.47–0.85) for all reading skills. Moreover, results demonstrated a significant interaction effect of grade X literacy group for word reading accuracy, *F*(3.83, 229.60) = 10.67, *p* < 0.001, η^2^_ρ_ = 0.15, word reading fluency, *F*(3.93, 235.59) = 6.99, *p* < 0.001, η^2^_ρ_ = 0.10, text-reading fluency, *F*(3.26, 195.85) = 21.06, *p* < 0.001, η^2^_ρ_ = 0.26, and reading comprehension, *F*(3.94, 236.19) = 47.51, *p* < 0.001, η^2^_ρ_ = 0.44^2^. Looking at Figure 2, it seems that the significant interactions might be the outcome of the different raw score growth of reading skills among the three literacy groups from Grade 1 to Grade 3.

Results from follow-up one-way ANOVAs with post hoc Bonferroni comparisons (see Table 4) showed that children with pRCD and children with rRCD showed significantly higher mean difference scores on word reading accuracy from Grade 1 to Grade 2 and from Grade 1 to Grade 3 than TD children. Furthermore, children with pRCD showed significantly lower mean difference scores on word reading fluency from Grade 2 to Grade 3 and from Grade 1 to Grade 3 than children with rRCD and TD children. The mean difference scores of children with pRCD on text-reading fluency were significantly lower than those of TD children across all three time intervals and of children with rRCD from Grade 2 to Grade 3 and from Grade 1 to Grade 3. Regarding reading comprehension, both children with pRCD and children with rRCD showed lower mean difference scores from Grade 1 to Grade 2 than TD children. However, although children with pRCD continued to significantly lag behind TD children on mean difference scores from Grade 2 to Grade 3, children with rRCD showed significantly higher mean difference scores than both groups of children. Finally, results showed that children with pRCD showed significantly lower mean difference scores from Grade 1 to Grade 3 than TD children and children with rRCD.

## 4. Discussion

The present longitudinal retrospective study examined the differences in the raw score growth of a broad range of oral language and reading skills from Grade 1 to Grade 3 among children identified in Grade 3 with persistent reading comprehension difficulties (pRCD), those exhibiting a resolving tendency of reading comprehension difficulties (rRCD), and typically developing (TD) peers. Overall, our results revealed significant differences across all the assessed skills, except from vocabulary, providing valuable information for intervention policy for RCD. Our findings are more extensively discussed below in correspondence to the formulated research hypotheses and previous research findings.

### 4.1. Differences in the Raw Score Growth of Oral Language Skills

In line with our first hypothesis, results showed that children with pRCD exhibited a slower raw score growth of MA than children with rRCD and TD children from Grade 1 to Grade 3. Notably, although children with pRCD and children with rRCD exhibited comparable levels of MA during the first two elementary grades, a more accelerated MA growth was associated with a resolving tendency of RCD by Grade 3. By enhancing their morphological awareness, children with RCD may become better equipped to exploit the semantic information conveyed by morphemes and to decode morphologically complex words more efficiently by segmenting them into their constituent morphemes—processes that collectively facilitate reading comprehension (Giazitzidou et al., 2025; Kieffer, 2014; Levesque et al., 2017). Within the Simple View of Reading framework, MA can be considered as a component of linguistic comprehension that facilitates the formation of high-quality lexical representations. Slower MA growth in pRCD children may reflect constraints in the linguistic resources available for reading comprehension. Also, it should be noted that although children with rRCD lagged behind TD children on MA at all time points, the two groups showed a similar MA raw score growth from Grade 1 to Grade 3. Thus, it could be suggested that the changes over time of MA for children with rRCD may be better described by a developmental delay (Stanovich et al., 1986), while that of children with pRCD may reflect a developmental deficit that might prevent them from adequately developing their reading comprehension (Francis et al., 1996).

On the other hand, our results did not reveal any significant differences on vocabulary raw score growth among the three literacy groups from Grade 1 to Grade 3; therefore, our first hypothesis was partially confirmed. In particular, both children with pRCD and children with rRCD consistently lagged behind TD children on vocabulary in Grades 1 and 2, corroborating that limited lexical knowledge is a risk factor for difficulties in reading comprehension (Catts et al., 2006; Grigorakis et al., 2022). However, prior research has indicated that improvements in vocabulary after an oral language intervention can mediate substantial gains in reading comprehension for children with RCD (Clarke et al., 2010). Accordingly, it was hypothesized that the significant advances in reading comprehension of children with rRCD from Grade 2 to Grade 3 would be associated with a higher raw score growth on vocabulary compared to children with pRCD. Although this was not confirmed by our results, children with rRCD outperformed their pRCD counterparts in vocabulary knowledge in Grade 3. Previous studies have suggested that the contribution of vocabulary on reading comprehension development becomes increasingly important as children get older (Ouellette & Beers, 2010; Storch & Whitehurst, 2002). Thus, the significant differences in vocabulary observed between the two groups in Grade 3 may foreshadow a more rapid vocabulary growth for children with rRCD in the subsequent elementary grades.

Regarding phonological processing skills, we found that both children with pRCD and children with rRCD showed more rapid changes in their phonological awareness levels from Grade 1 to Grade 3 than TD children. However, only children with rRCD showed a faster raw score growth of RAN than TD children; therefore, our second hypothesis was partially confirmed. More specifically, although children with pRCD and children with rRCD showed similar PA levels in Grade 1, they were both significantly outperformed by TD children. Despite their initial PA deficits, children with pRCD and children with rRCD developed PA at a faster rate than TD children already from Grade 1. It is noteworthy that despite the inclusion of more demanding items in the administered PA tasks in Grade 3, the two groups showed a similar raw score growth with TD children from Grade 2 to Grade 3. Thus, our results confirm prior research indicating that in transparent orthographies children with literacy difficulties manage to adequately develop their PA skills, due to the high consistency in the grapheme-phoneme correspondences (Landerl & Wimmer, 2000). However, despite the significant improvements on PA, both children with pRCD and children with rRCD continued to lag behind TD children in Grade 3. Moreover, another interesting finding is that although children with pRCD and children with rRCD showed similar PA levels in Grade 1 and similar PA growth rate between Grades 1 and 3, children with rRCD significantly outperformed children with pRCD on PA in Grades 1 and 2. These findings might advocate in favor of an implicit association of PA with reading comprehension development in line with previous research evidence (Georgiou et al., 2009; Høien-Tengesdal, 2010; Kargiotidis et al., 2022).

Moreover, our results showed that children with pRCD did not differ from the other two groups in the raw score growth of RAN from Grade 1 to Grade 3. Furthermore, their performance on the RAN tasks was similar to that of children with rRCD across grades, with both groups consistently underperforming compared to TD children. However, children with rRCD showed a steeper raw score growth of RAN than TD children from Grade 1 to Grade 2 and overall from Grade 1 to Grade 3, slightly reducing their initial deficits. Research findings from intervention studies indicate that improvements in RAN can be translated into gains in reading comprehension (Wolfsperger & Mayer, 2024) or reading accuracy and fluency (Margheri et al., 2025; Pecini et al., 2019). Thus, our findings might provide preliminary evidence implying that the faster raw score growth of RAN exhibited by children with rRCD might be associated with the development of reading comprehension either directly or indirectly through the enhancement of reading fluency.

### 4.2. Differences in the Raw Score Growth of Reading Skills

In line with our third hypothesis, we found that both children with pRCD and children with rRCD showed a steeper raw score growth in word reading accuracy than TD children from Grade 1 to Grade 3. Already by the beginning of Grade 2, both groups started to diminish their initial deficits and gradually approaching the performance of TD children. Our findings corroborated prior research in transparent orthographies, indicating that children with reading difficulties, despite early challenges in word decoding, tend to develop adequate word reading accuracy relatively early (Aro & Wimmer, 2003; Landerl & Wimmer, 2008). However, both groups of children continued to lag behind TD children on word reading accuracy levels in Grade 3. In their study with Greek-speaking children, Constantinidou and Stainthorp (2009) found that children with reading difficulties were less accurate in word reading than their chronological age-matched peers and that TD children managed to achieve ceiling scores in word reading accuracy by the age of nine. Similarly, in our study, TD children were approaching ceiling performance at the end of Grade 3. Thus, the slower raw score growth observed in TD children may be attributed to the high consistency of Greek orthography, which enables the rapid attainment of a very high level of word reading accuracy by Greek students as early as Grade 2—leaving no room for further improvement (Seymour et al., 2003). However, it is, also, of particular interest that although children with pRCD and children with rRCD showed similar word reading accuracy levels in Grade 1 and similar raw score growth between Grade 1 and Grade 3, children with rRCD performed significantly better on word reading accuracy in Grades 2 and 3. One possible explanation could be that the ability of children with rRCD to read words more accurately is not associated with a higher growth rate because of the trivial role of word reading accuracy in the prediction of reading comprehension difficulties in Greek orthography (Kargiotidis et al., 2025).

Finally, our results demonstrated that children with rRCD developed their word reading fluency and text-reading fluency at a more accelerated rate than children with pRCD from Grade 1 to Grade 3, corroborating both the fourth and the fifth hypotheses of the present study. It is noteworthy that children with rRCD started to develop both word reading fluency and text reading fluency at a faster rate after Grade 2, which coincides with the more rapid reading comprehension development. Several studies have consistently shown that reading fluency, both on word and text-level, is a stronger determinant of reading comprehension development and a more reliable predictor of difficulties in reading comprehension than word reading accuracy (Capin et al., 2022; Kang & Shin, 2019; Kargiotidis et al., 2025). Our study adds on this prior research evidence by indicating that substantial gains on reading fluency for children with reading comprehension difficulties might be associated with reading comprehension development. Furthermore, it is of particular interest that based on the effect sizes, our findings showed that the differences between the two groups of children were larger on the raw score growth of text-reading fluency than on word reading fluency. These findings provide additional support to previous research indicating that after Grade 1 and as children grow older, the role of text-reading fluency on reading comprehension development becomes more prominent (Clemens et al., 2017; Eason et al., 2013; Y. G. Kim & Wagner, 2015).

Overall, the findings of the present study showed that the primary developmental differences between the two groups of children with RCD were the more increased raw score growth displayed by children with rRCD in morphological awareness, word reading fluency and text-reading fluency. Although both groups showed similar skill levels in Grade 1, children with rRCD gradually narrowed their early deficits and outperformed children with pRCD by the end of Grade 3. These patterns can be interpreted within the Simple View of Reading framework as describing differences in the balance between decoding efficiency and linguistic comprehension. Children with rRCD may benefit from a more dynamic interaction of these components, enabling gradual improvements in reading comprehension despite early deficits. Finally, compared to their TD peers, both RCD groups continued to lag behind across nearly all measures, despite exhibiting a more accelerated rate in phonological processing skills and word reading accuracy, possibly, due to ceiling effects. The present findings are consistent with the notion of protracted response patterns in poor responders to instruction. Children with pRCD exhibited consistently weaker performance across most oral language and reading measures from Grade 1 to Grade 3, reflecting a slower and more constrained raw score growth that may indicate persistent deficits limiting their capacity to benefit from typical instruction. In contrast, children with rRCD, despite initial difficulties, showed more accelerated raw score growth in morphological awareness, word reading fluency, and text-reading fluency, suggesting greater sensitivity to various aspects of oral and written language structure even in Grade 1. This early sensitivity likely facilitated more efficient acquisition of advanced reading and language skills over time, allowing the rRCD group to gradually narrow their deficits relative to children with pRCD.

## 5. Limitations and Future Research

Although the present study employed a longitudinal research design and comprehensively examined the raw score growth of several oral language and reading skills, it is subject to specific limitations that could guide future research. First, reading comprehension difficulties were identified using certain diagnostic criteria that required a performance below the 25th percentile on a standardized reading comprehension measure in Grades 1 and 2. Reading comprehension difficulties were considered to be persistent when children continued to perform below the 25th percentile in Grade 3. On the other hand, a performance above the 37th percentile in Grade 3 was indicative of a resolving tendency of RCD, in line with prior research using a similar cut-off score to identify typical reading comprehension performance (Bailey et al., 2016; Kelso et al., 2020). However, as children get older written texts become more complex in sentence structure, vocabulary, and conceptual content, rendering reading comprehension a more cognitively demanding task (Cain, 2016). Thus, future studies could extend our research by examining whether the particular gains exhibited by children with rRCD are maintained in the upper elementary grades.

Second, although our findings may imply that substantial growth in particular oral language and reading skills may assist children with RCD in enhancing their reading comprehension—and possibly overcome their difficulties—they do not allow causal interpretations to be made. One of the main reasons is that in the present study no information was collected regarding the type, intensity, or quality of literacy instruction received by children with RCD. Also, the underpowered sample size, especially of the rRCD group, did not allow us to conduct more advanced statistical analysis (e.g., multi-level growth modeling) that could clarify further these complex relationships. Thus, although our findings are being discussed in line with previous experimental research, they cannot establish causal pathways. Research findings from previous intervention studies may provide a preliminary basis for a causal association (Goodwin & Ahn, 2010; Stevens et al., 2016); however, additional research is required to draw safer conclusions.

Third, the present study did not control for specific cognitive skills, such as working memory, which contribute to higher-level text processing, and thus are closely associated with reading comprehension (C. A. Perfetti et al., 1996). Thus, future research should incorporate assessments of working memory and other relative cognitive skills to clarify whether the observed developmental patterns can be attributed exclusively to the oral language and reading skills examined.

Fourth, assessing vocabulary only with an expressive vocabulary measure might explain the lack of a significant difference on its growth rate between children with pRCD and children with rRCD. Empirical research has shown that the predictive role of vocabulary on reading comprehension is stronger when it is assessed with both an expressive and a receptive measure (Protopapas et al., 2013). Thus, future research could examine both vocabulary aspects in an attempt to further elucidate our findings.

Lastly, a further limitation concerns the measurement equivalence of several constructs across grades. Although the same underlying skills were assessed at each time point, some tasks (e.g., those for phonological awareness and morphological awareness) used grade-specific item sets to avoid floor and ceiling effects. As a result, strict measurement invariance cannot be assumed, and part of the observed developmental change may reflect differences in task difficulty. Therefore, interpretations of developmental changes should be made with caution.

## 6. Implications for Practice

Our findings have important implications for educational practice and intervention policy, providing useful guidance to teachers and other professionals who support children with reading comprehension difficulties. In particular, in line with previous studies (Grigorakis et al., 2022; Kargiotidis et al., 2025), our results indicate that as early as Grade 1 children with deficits across multiple oral language and reading skills are at great risk of developing reading comprehension difficulties in later grades. Thus, incorporating comprehensive early screening procedures can serve as a valuable tool on teachers’ efforts to accurately identify children at risk for difficulties in reding comprehension and implement timely educational interventions.

Regarding intervention policy, previous studies have shown that morphological instruction (Goodwin & Ahn, 2010) and connected-text reading practice (Stevens et al., 2016) can be rather beneficial for children with literacy difficulties and facilitate the development of reading comprehension. The present study extends this literature by demonstrating that a faster growth in both MA and reading fluency can be associated with significant improvements in reading comprehension of children with reading comprehension difficulties. Thus, our findings suggest that, in order to maximize the impact of educational interventions, they should integrate morphological instruction with connected-text reading practice. For instance, teachers can provide brief, explicit learning activities on prefixes, suffixes, and roots and then integrate them in connected texts. This may include highlighting morphemes in a reading passage, classifying words into categories based on morphological structure, or motivating students to analyze unknown words using morphological clues while reading.

Finally, a closer examination of the effect sizes of the difference scores on oral language skills between the three literacy groups reveals a developmental shift from phonologically oriented to semantically oriented skills across grades (Vellutino et al., 2004). These findings highlight the need for teachers to gradually enrich initial phonological decoding instruction with educational practices that promote the development of morphological awareness and vocabulary. For example, teachers can associate decoding activities to meaning by highlighting common affixes during word-reading practice. Also, vocabulary instruction can include expressive and receptive activities, such as previewing key words before reading, using semantic mapping, and modeling how to extract meaning from context and morphology during reading. By implementing these educational practices, they will be able to more efficiently address the evolving linguistic demands of reading comprehension across the elementary grades.

## Figures and Tables

**Table 1:** Means (M) and standard deviations (SD) for reading measures assessed in Grades 1, 2, and 3 for the three literacy groups.

Measures	pRCD Group		rRCD Group		TD Group		ANOVA *F*-Test df = 2, 120
	M	SD	M	SD	M	SD	
Word reading accuracy							
Grade 1	22.88 ^1^	12.56	25.75 ^1^	9.04	42.86 ^2^	7.48	57.52 ***, η^2^_ρ_ = 0.49
Grade 2	31.24 ^1^	11.92	37.56 ^2^	8.28	45.81 ^3^	5.30	35.98 ***, η^2^_ρ_ = 0.38
Grade 3	39.71 ^1^	8.99	46.88 ^2^	4.46	52.24 ^3^	3.55	51.14 ***, η^2^_ρ_ = 0.46
Word reading fluency							
Grade 1	14.98 ^1^	7.16	19.00 ^1^	7.37	31.57 ^2^	10.28	49.39 ***, η^2^_ρ_ = 0.45
Grade 2	22.55 ^1^	8.46	28.50 ^1^	8.53	39.84 ^2^	12.70	35.45 ***, η^2^_ρ_ = 0.37
Grade 3	36.69 ^1^	10.79	50.13 ^2^	9.72	58.28 ^3^	11.33	52.00 ***, η^2^_ρ_ = 0.46
Text-reading fluency							
Grade 1	20.33 ^1^	9.77	24.81 ^1^	8.71	46.41 ^2^	18.01	48.01 ***, η^2^_ρ_ = 0.44
Grade 2	30.61 ^1^	12.44	41.75 ^1^	12.34	62.47 ^2^	20.98	47.36 ***, η^2^_ρ_ = 0.44
Grade 3	59.73 ^1^	19.39	85.81 ^2^	18.60	110.59 ^3^	24.99	70.07 ***, η^2^_ρ_ = 0.54
Reading comprehension ^4^							
Grade 1	6.41 ^1^	4.80	7.44 ^1^	5.07	14.93 ^2^	0.77	82.94 ***, η^2^_ρ_ = 0.58
Grade 2	10.29 ^1^	2.80	11.13 ^1^	1.71	25.03 ^2^	5.25	195.72 ***, η^2^_ρ_ = 0.77
Grade 3	17.71 ^1^	3.85	29.69 ^2^	3.61	35.84 ^3^	3.69	313.17 ***, η^2^_ρ_ = 0.84

*Note.* ^1,2,3^ Superscript numbers refer to pairwise comparisons (Bonferroni). Means with the same superscript number do not differ significantly. ^4^ Reading comprehension was assessed with a different measure in Grade 1 compared to Grades 2 and 3. pRCD = Persistent reading comprehension difficulties; rRCD = Resolving tendency of reading comprehension difficulties; TD = Typically developing. *** *p* < 0.001.

**Table 2:** Means (M) and standard deviations (SD) for non-verbal intelligence in Grade 1 and oral language measures assessed in Grades 1, 2, and 3 for the three literacy groups.

Measures	pRCD Group		rRCD Group		TD Group		ANOVA *F*-Test df = 2, 120
	M	SD	M	SD	M	SD	
Non-verbal intelligence							
Grade 1	17.41 ^1^	3.30	17.50 ^1^	2.68	22.17 ^2^	4.08	26.17 ***, η^2^_ρ_ = 0.30
Phonological awareness ^4^							
Grade 1	25.47 ^1^	11.56	31.81 ^1^	11.30	55.55 ^2^	11.88	93.16 ***, η^2^_ρ_ = 0.61
Grade 2	42.06 ^1^	11.90	52.06 ^2^	10.50	65.81 ^3^	6.87	81.32 ***, η^2^_ρ_ = 0.58
Grade 3	72.37 ^1^	15.94	82.25 ^2^	11.25	95.74 ^3^	6.50	53.15 ***, η^2^_ρ_ = 0.47
Morphological awareness ^4^							
Grade 1	8.59 ^1^	5.42	9.06 ^1^	6.30	20.66 ^2^	7.54	50.03 ***, η^2^_ρ_ = 0.46
Grade 2	13.08 ^1^	5.79	16.25 ^1^	6.11	28.98 ^2^	4.54	127.86 ***, η^2^_ρ_ = 0.68
Grade 3	22.90 ^1^	7.28	28.50 ^2^	7.18	39.16 ^3^	3.98	101.85 ***, η^2^_ρ_ = 0.63
Vocabulary							
Grade 1	10.76 ^1^	3.27	12.44 ^1^	4.56	15.90 ^2^	3.21	30.55 ***, η^2^_ρ_ = 0.34
Grade 2	13.16 ^1^	3.39	14.63 ^1^	3.67	18.86 ^2^	3.57	36.53 ***, η^2^_ρ_ = 0.38
Grade 3	15.92 ^1^	3.43	18.88 ^2^	2.06	22.50 ^3^	3.20	57.22 ***, η^2^_ρ_ = 0.49
Rapid automatized naming							
Grade 1	23.421 ^1^	4.681	23.502 ^1^	6.692	18.553 ^2^	3.494	18.16 ***, η^2^_ρ_ = 0.23
Grade 2	19.533 ^1^	3.503	18.153 ^1^	2.886	15.938 ^2^	2.655	18.71 ***, η^2^_ρ_ = 0.24
Grade 3	17.890 ^1^	3.247	16.568 ^1^	2.350	14.604 ^2^	2.466	18.59 ***, η^2^_ρ_ = 0.24

*Note.* ^1,2,3^ Superscript numbers refer to pairwise comparisons (Bonferroni). Means with the same superscript number do not differ significantly. ^4^ Maximum score in Grades 1 and 2 differs from the maximum score in Grade 3 due to differences in the number of items in the respective component tasks. pRCD = Persistent reading comprehension difficulties; rRCD = Resolving tendency of reading comprehension difficulties; TD = Typically developing. *** *p* < 0.001.

**Table 3:** Means (M) and standard deviations (SD) of the difference scores between the three grades for oral language skills across literacy groups.

Measures	pRCD Group		rRCD Group		TD Group		ANOVA *F*-Test df = 2, 120	Post Hoc Comparisons *t*-Test
	M	SD	M	SD	M	SD		
Phonological awareness ^3^								
Grade 2–Grade 1	16.59 ^1^	10.70	20.25 ^1^	9.68	10.26 ^2^	8.01	10.04 ***, η^2^_ρ_ = 0.14	pRCD vs. TD, 3.48 **, *d* = 0.68 rRCD vs. TD, 3.77 ***, *d* = 1.19
Grade 3–Grade 2	30.31 ^1^	13.31	30.19 ^1^	8.17	29.93 ^1^	6.23	0.02, η^2^_ρ_ = 0	
Grade 3–Grade 1	46.90 ^1^	15.69	50.44 ^1^	9.49	40.19 ^2^	9.47	6.28 **, η^2^_ρ_ = 0.10	pRCD vs. TD, 2.80 *, *d* = 0.53 rRCD vs. TD, 2.94 *, *d* = 1.08
Morphological awareness ^3^								
Grade 2–Grade 1	4.49 ^1^	5.94	7.19 ^1,2^	7.40	8.33 ^2^	5.30	5.77 **, η^2^_ρ_ = 0.09	pRCD vs. TD, 3.38 **, *d* = 0.69
Grade 3–Grade 2	9.82 ^1^	5.94	12.25 ^1^	5.86	10.17 ^1^	3.63	1.48, η^2^_ρ_ = 0.02	
Grade 3–Grade 1	14.31 ^1^	7.49	19.44 ^2^	6.75	18.50 ^2^	6.25	6.22 **, η^2^_ρ_ = 0.09	pRCD vs. rRCD, 2.61 *, *d* = 0.70 pRCD vs. TD, 3.16 **, *d* = 0.61
Rapid automatized naming								
Grade 2–Grade 1	−3.89 ^1,2^	3.26	−5.35 ^1^	5.45	−2.62 ^2^	2.33	5.09 **, η^2^_ρ_ = 0.08	rRCD vs. TD, 2.98 *, *d* = 0.84
Grade 3–Grade 2	−1.64 ^1^	2.26	−1.58 ^1^	2.54	−1.33 ^1^	1.87	0.30, η^2^_ρ_ = 0.01	
Grade 3–Grade 1	−5.53 ^1,2^	3.43	−6.93 ^1^	6.19	−3.95 ^2^	2.66	5.35 **, η^2^_ρ_ = 0.08	rRCD vs. TD, 2.95 *, *d* = 0.81

*Note.* ^1,2^ Superscript numbers refer to pairwise comparisons (Bonferroni). Means with the same superscript number do not differ significantly. ^3^ Maximum score in Grades 1 and 2 differs from the maximum score in Grade 3 due to differences in the number of items in the respective component tasks. pRCD = Persistent reading comprehension difficulties; rRCD = Resolving tendency of reading comprehension difficulties; TD = Typically developing. *** *p* < 0.001, ** *p* < 0.01, * *p* < 0.05.

**Table 4:** Means (M) and standard deviations (SD) of the difference scores between the three grades for reading skills across literacy groups.

Measures	pRCD Group		rRCD Group		TD Group		ANOVA *F*-Test df = 2, 120	Post-Hoc Comparisons *t*-Test
	M	SD	M	SD	M	SD		
Word reading accuracy								
Grade 2–Grade 1	8.37 ^1^	9.21	11.81 ^1^	8.79	2.95 ^2^	6.83	10.25 ***, η^2^_ρ_ = 0.15	pRCD vs. TD, 3.45 **, *d* = 0.68 rRCD vs. TD, 3.87 ***, *d* = 1.22
Grade 3–Grade 2	8.47 ^1^	8.56	9.31 ^1^	6.92	6.43 ^1^	4.56	1.81, η^2^_ρ_ = 0.03	
Grade 3–Grade 1	16.84 ^1^	10.35	21.13 ^1^	8.62	9.38 ^2^	6.85	16.42 ***, η^2^_ρ_ = 0.22	pRCD vs. TD, 4.46 ***, *d* = 0.86 rRCD vs. TD, 4.82 ***, *d* = 1.62
Word reading fluency								
Grade 2–Grade 1	7.57 ^1^	5.02	9.50 ^1^	5.23	8.28 ^1^	7.93	0.54, η^2^_ρ_ = 0.01	
Grade 3–Grade 2	14.14 ^1^	6.51	21.63 ^2^	5.23	18.43 ^2^	7.89	8.60 ***, η^2^_ρ_ = 0.13	pRCD vs. rRCD, 3.68 **, *d* = 1.20 pRCD vs. TD, 3.13 **, *d* = 0.59
Grade 3–Grade 1	21.71 ^1^	8.63	31.13 ^2^	7.06	26.71 ^2^	7.61	10.13 ***, η^2^_ρ_ = 0.14	pRCD vs. rRCD, 4.10 ***, *d* = 1.14 pRCD vs. TD, 3.23 **, *d* = 0.62
Text-reading fluency								
Grade 2–Grade 1	10.29 ^1^	7.80	16.94 ^1,2^	7.79	16.05 ^2^	14.03	4.21 *, η^2^_ρ_ = 0.07	pRCD vs. TD, 2.65 *, *d* = 0.50
Grade 3–Grade 2	29.12 ^1^	12.99	44.06 ^2^	13.08	48.12 ^2^	16.68	22.36 ***, η^2^_ρ_ = 0.27	pRCD vs. TD, 6.59 ***, *d* = 1.26 pRCD vs. rRCD, 3.49 **, *d* = 1.15
Grade 3–Grade 1	39.41 ^1^	16.61	61.00 ^2^	14.78	64.17 ^2^	19.70	26.73 ***, η^2^_ρ_ = 0.31	pRCD vs. TD, 7.11 ***, *d* = 1.35 pRCD vs. rRCD, 4.18 ***, *d* = 1.33
Reading comprehension ^4^								
Grade 2–Grade 1	3.88 ^1^	4.40	3.69 ^1^	5.70	10.10 ^2^	5.08	25.03 ***, η^2^_ρ_ = 0.29	pRCD vs. TD, 6.54 ***, *d* = 1.30 rRCD vs. TD, 4.63 ***, *d* = 1.23
Grade 3–Grade 2	7.43 ^1^	3.75	18.56 ^2^	3.85	10.81 ^3^	4.67	42.34 ***, η^2^_ρ_ = 0.41	pRCD vs. TD, 4.13 ***, *d* = 0.79 rRCD vs. pRCD, 9.16 ***, *d* = 2.95 rRCD vs. TD, 6.51 ***, *d* = 1.72
Grade 3–Grade 1	11.31 ^1^	4.71	22.25 ^2^	4.01	20.91 ^2^	3.67	84.18 ***, η^2^_ρ_ = 0.58	pRCD vs. TD, 11.91 ***, *d* = 2.30 pRCD vs. rRCD, 9.14 ***, *d* = 2.40

*Note.* ^1,2,3^ Superscript numbers refer to pairwise comparisons (Bonferroni). Means with the same superscript number do not differ significantly. ^4^ Reading comprehension was assessed with a different measure in Grade 1 compared to Grades 2 and 3. pRCD = Persistent reading comprehension difficulties; rRCD = Resolving tendency of reading comprehension difficulties; TD = Typically developing. *** *p* < 0.001, ** *p* < 0.01, * *p* < 0.05.

## Data Availability

Data is unavailable due to privacy and ethical restrictions.

## Notes

1 Mauchly’s test of sphericity was significant (*p* < 0.05) for phonological awareness, morphological awareness, and RAN. The value of Greenhouse-Geiser *ε* was above 0.75 for all of them. Thus, we applied the Huynh-Feldt correction (Field, 2018).

2 Mauchly’s test of sphericity was significant (*p* < 0.05) for word reading accuracy, word reading fluency, text-reading fluency, and reading comprehension. The value of Greenhouse-Geiser *ε* was above 0.75 for all of them. Thus, we applied the Huynh-Feldt correction (Field, 2018).

## Abbreviations

The following abbreviations are used in this manuscript:

TD	Typically developing children
pRCD	Persistent reading comprehension difficulties
rRCD	Resolving tendency of reading comprehension difficulties
RCD	Reading comprehension difficulties
RAN	Rapid automatized naming
PA	Phonological awareness
MA	Morphological awareness
